# Author Correction: Structural dynamics of AAA + ATPase Drg1 and mechanism of benzo-diazaborine inhibition

**DOI:** 10.1038/s41467-023-36072-4

**Published:** 2023-01-27

**Authors:** Chengying Ma, Damu Wu, Qian Chen, Ning Gao

**Affiliations:** 1 State Key Laboratory of Membrane Biology, Peking-Tsinghua Joint Center for Life Sciences, School of Life Sciences, Peking University, 100871 Beijing, China; 2 Changping Laboratory, 102206 Beijing, China; 3 National Biomedical Imaging Center, Peking University, 100871 Beijing, China

**Keywords:** Cryoelectron microscopy, Ribosome, Chaperones, Target validation

Correction to: *Nature Communications* 10.1038/s41467-022-34511-2, published online 09 November 2022

The original version of this Article contained errors in the Results section.

The first incorrect sentence read ‘The residues 29–230, 244–509, and 515–777 constitute the NTD, D1, and D2, respectively.’ The correct version states ‘The residues **29–288**, 250–499, and 521–77 constitute the NTD, D1, and D2, respectively. ’

The second incorrect sentence read ‘In addition, a few 3D classes also show highly flexible D2 rings (Supplementary Figs. 3c and 4c).’ The correct version states ‘(Supplementary Figs. 2a, 3a, 4a)’ in place of ‘(Supplementary Figs. 3c and 4c)’.

The third incorrect sentence read ‘The PLs (PL-I and PL-II) form upward spiral steps around the peptide and display an anticlockwise rotation pattern viewed from the D1 ring.’ The correct version states ‘D2’ in place of ‘D1’.

The fourth incorrect sentence read ‘Based on the structural analysis, we introduced four separate mutations (R499A, M503A, R504A, and D507A) to the D1-D2 linker of Drg1.’ The correct version states ‘ F507A’ in place of ‘D507A’.

The fifth incorrect sentence read ‘Unlike the NTD-D1 linker, the D1-D2 linker of Drg1 is highly conserved (Fig. 4a).’ The correct version states ‘ (Supplementary Fig. 11c)’ in place of ‘(Fig. 4a)’.

These have been corrected in both the PDF and HTML versions of the Article.

