# Supplementary material for: Structural dynamics of AAA + ATPase Drg1 and mechanism of benzo-diazaborine inhibition
Source: Nat Commun. 2022 Nov 9;13:6765. doi: 10.1038/s41467-022-34511-2 (PMC9646744; doi:10.1038/s41467-022-34511-2)
Supplement: Supplementary file 1 — Supplementary Information [file 41467_2022_34511_MOESM1_ESM.pdf]

Supplementary Information

Structural dynamics of AAA+ ATPase Drg1 and mechanism of benzo-diazaborine inhibition

Chengying Ma[1,2,4], Damu Wu[1,4], Qian Chen[1] and Ning Gao[1, 2, 3*]

Affiliations:

[1] State Key Laboratory of Membrane Biology, Peking-Tsinghua Joint Center for Life Sciences, School of Life Sciences, Peking University, Beijing 100871, China.

[2] Changping Laboratory, Beijing 102206, China

[3] National Biomedical Imaging Center, Peking University, Beijing 100871, China

[4] These authors contributed equally

*Correspondence to: gaon@pku.edu.cn

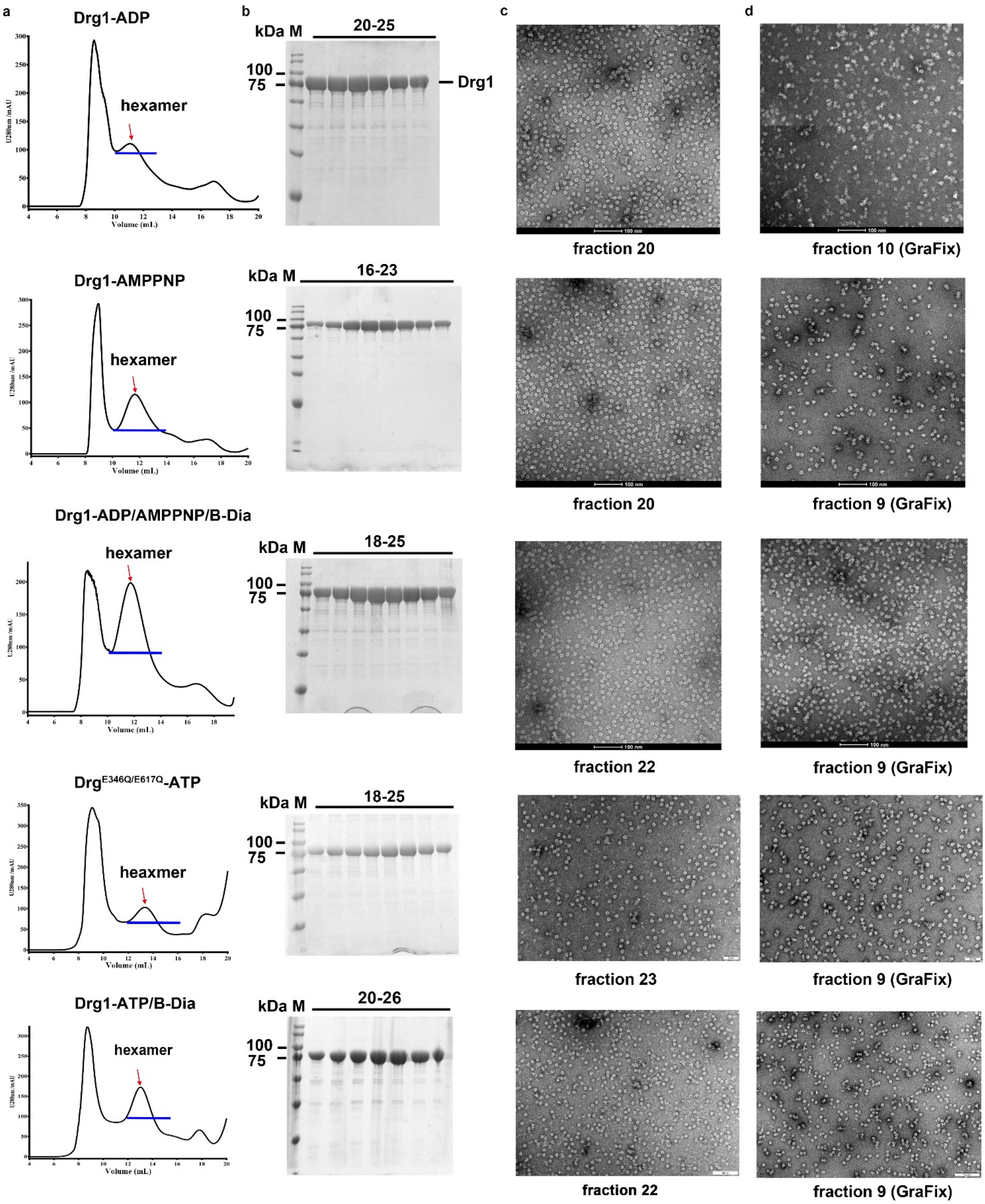

Supplementary Fig. 1 Sample preparation of Drg1 hexamers in different nucleotide or benzo-diazaborine binding states.

a, Purification of Drg1 hexamers in the presence ADP, AMPPNP, ADP/AMPPNP/B-Dia, ATP/B-Dia or ATP (for mutant Drg1$^{E346Q/E617Q}$) using size-exclusion chromatography. Hexamer fractions are indicated and labeled with arrows.

b, SDS-PAGE analysis of the hexamer fractions in **(a)**.

c, Negative staining electron microscopy of the peak fraction of the hexamers in **(a)**.

d, Negative staining electron microscopy of the hexamer fraction after the GraFix analysis.

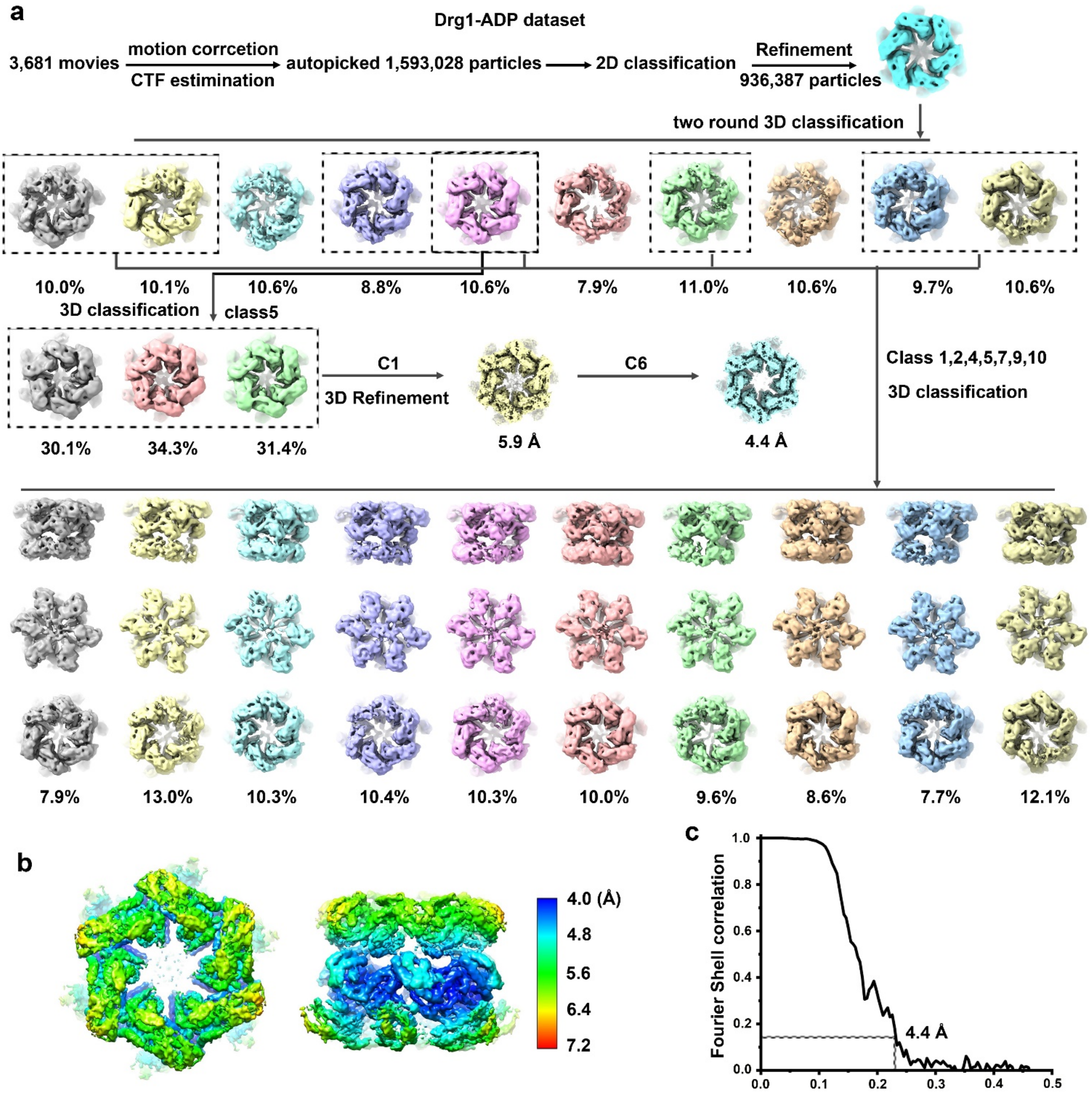

Supplementary Fig. 2 Image processing workflow of the Drg1-ADP dataset.

a, Image processing workflow of the Drg1-ADP dataset. The autopicked particles were subjected to multiple rounds of 2D and 3D classifications. Both the C1 and C6 symmetry were tested in the final refinement.

b-c, Local resolution estimation **(b)** and Fourier shell correlation (FSC) curves **(c)** of the final cryo-EM map (C6). The resolution was determined at the gold-standard FSC 0.143 criteria.

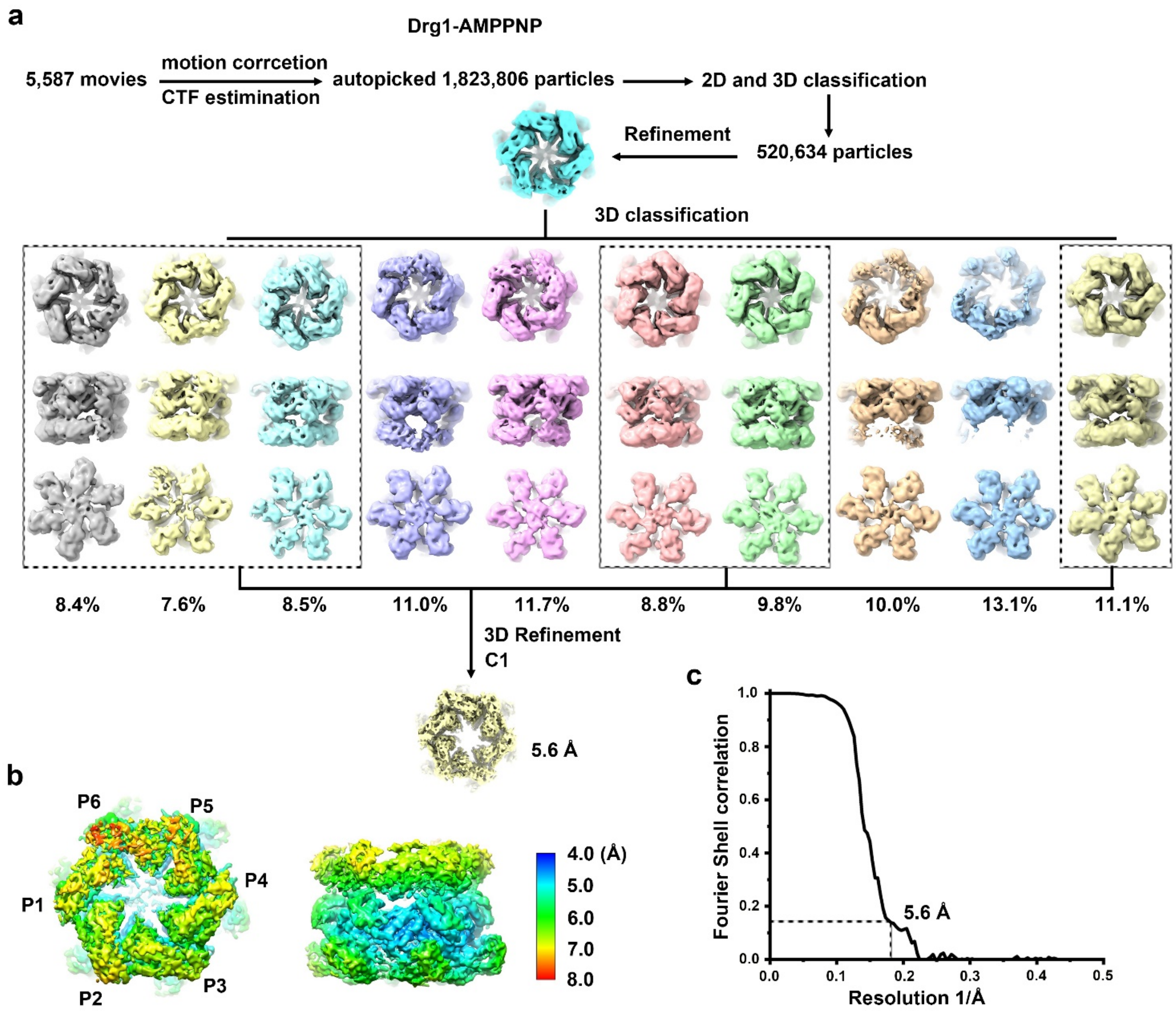

Supplementary Fig. 3 Image processing workflow of the Drg1-AMPPNP dataset.

a, Image processing workflow of the Drg1-AMPPNP dataset. The autopicked particles were subjected to multiple rounds of 2D and 3D classifications.

b-c, Local resolution estimation **(b)** and Fourier shell correlation (FSC) curves **(c)** of the final cryo-EM map (C1). The resolution was determined at the gold-standard FSC 0.143 criteria.

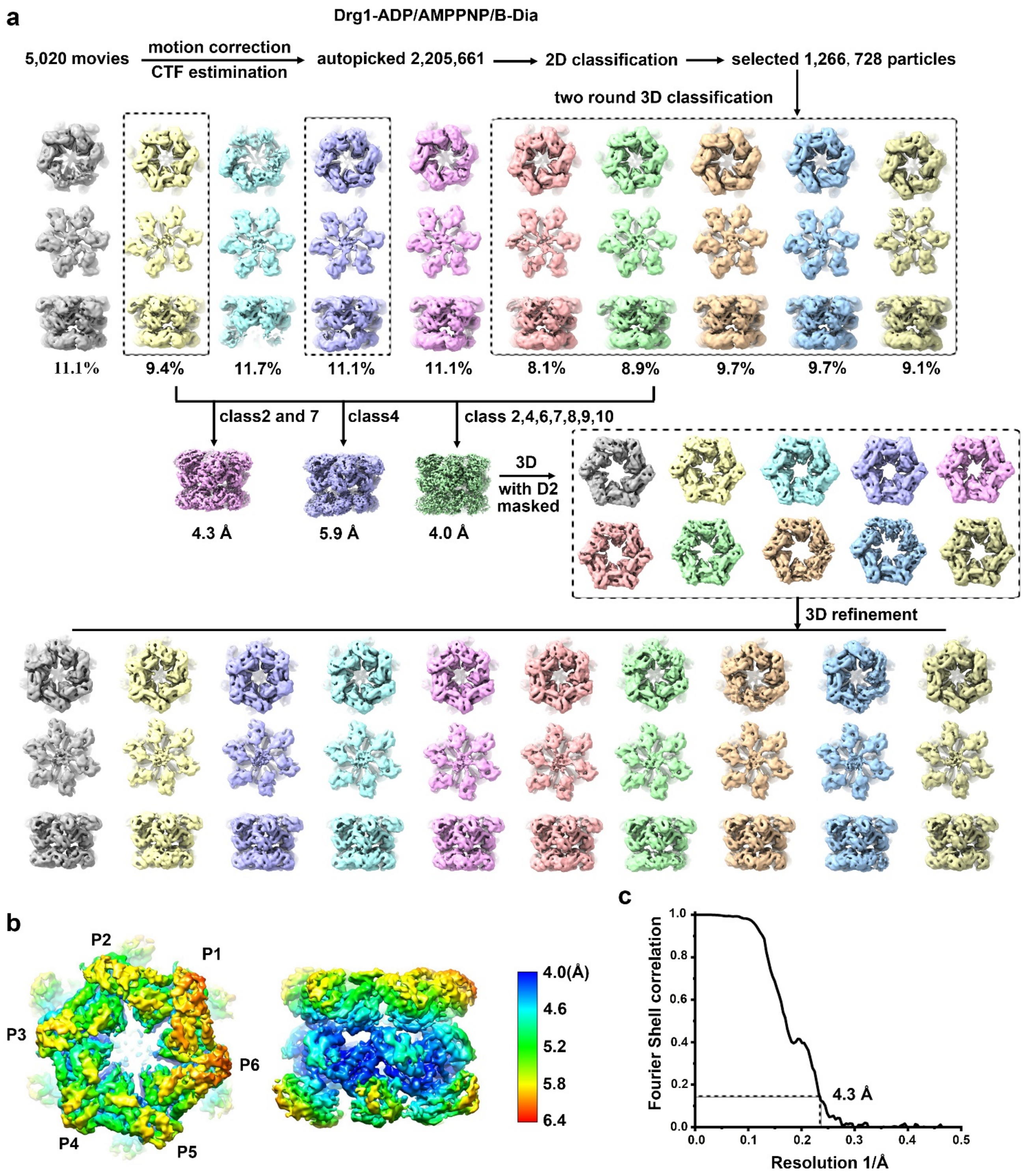

Supplementary Fig. 4 Image processing workflow of the Drg1-ADP/AMPPNP/B-Dia dataset.
a, Image processing workflow of the Drg1-ADP/AMPPNP/B-Dia dataset. The autopicked particles were subjected to multiple rounds of 2D and 3D classifications.
b-c, Local resolution estimation **(b)** and Fourier shell correlation (FSC) curves **(c)** of the final cryo-EM map (C1). The resolution was determined at the gold-standard FSC 0.143 criteria.

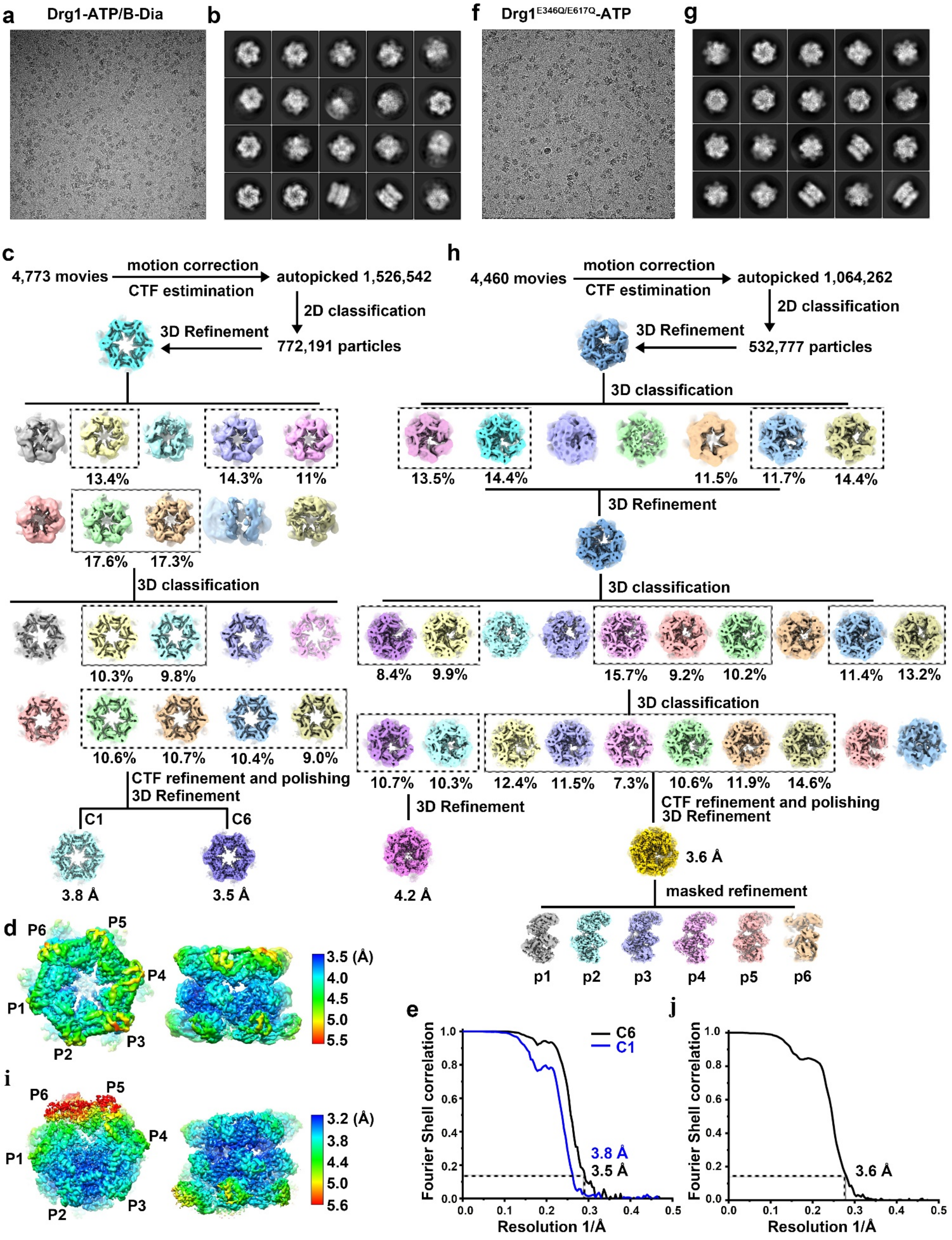

Supplementary Fig. 5 Image processing workflow of the Drg1-ATP/B-Dia and Drg1$^{E346Q/E617Q}$-ATP datasets.

a, A representative cryo-EM micrograph of the Drg1-ATP/B-Dia particles.

b, Representative 2D class averages of the Drg1-ATP/B-Dia particles.

c, Image processing workflow of the Drg1-ATP/B-Dia dataset. The autopicked particles were subjected to multiple rounds of 2D and 3D classifications. Both C1 and C6 symmetry were tested in the refinement.

d-e, Local resolution estimation **(d)** and Fourier shell correlation (FSC) curves **(e)** of the final cryo-EM map

of the Drg1-ATP/B-Dia complex (C6). The resolution was determined at the gold-standard FSC 0.143 criteria.
f, A representative cryo-EM micrograph of the mutant Drg1-ATP particles.
g, Representative 2D class averages of the mutant Drg1-ATP particles.
h, Image processing workflow of the mutant Drg1-ATP dataset. The autopicked particles were subjected to multiple rounds of 2D and 3D classifications.
i-j, Local resolution estimation **(i)** and Fourier shell correlation (FSC) curves **(j)** of the final cryo-EM map of th e mutant Drg1-ATP complex. The resolution was determined at the gold-standard FSC 0.143 criteria.

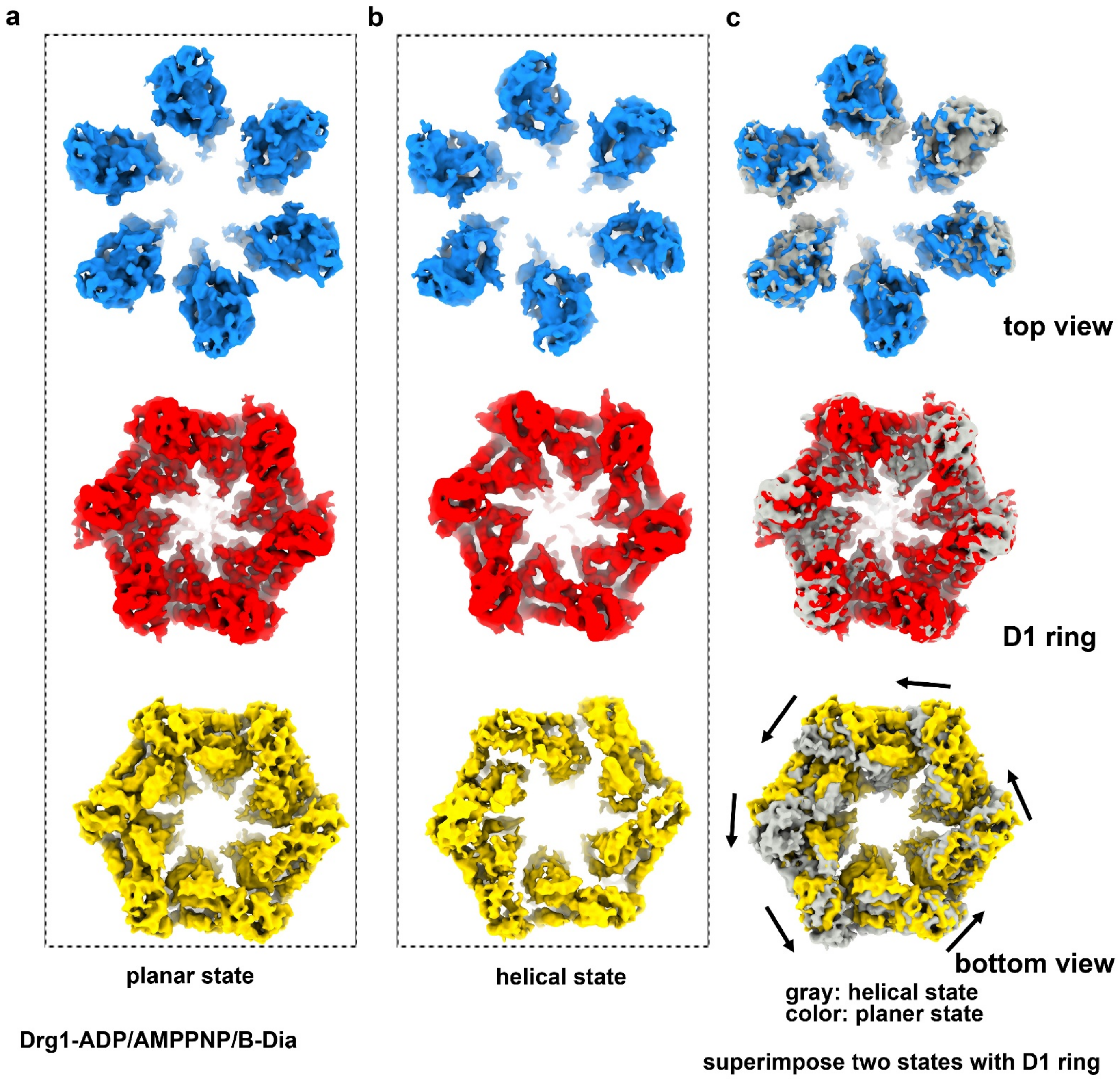

Supplementary Fig. 6 Structural comparison of planar and helical states of the Drg1 hexamers from the ADP/AMPPNP/B-Dia dataset.

a, Cryo-EM map of the NTD (top), D1 (middle) and D2 (bottom) rings of the Drg1 hexamer in the planar state from the Drg1-ADP/AMPPNP/B-Dia dataset.

b, Cryo-EM map of the NTD (top), D1 (middle) and D2 (bottom) rings of the Drg1 hexamer in the helical state from the Drg1-ADP/AMPPNP/B-Dia dataset.

c, Comparison of the NTD (top), D1 (middle) and D2 (bottom) rings of the planar and helical states. The two hexamer maps are aligned using the D1 rings as reference. Six subunits in the planar state are arranged more symmetrical while the subunits in the helical state adopt a spiral configuration. A large rotation of the D2 ring between the planar and helical states is shown and indicated by arrows.

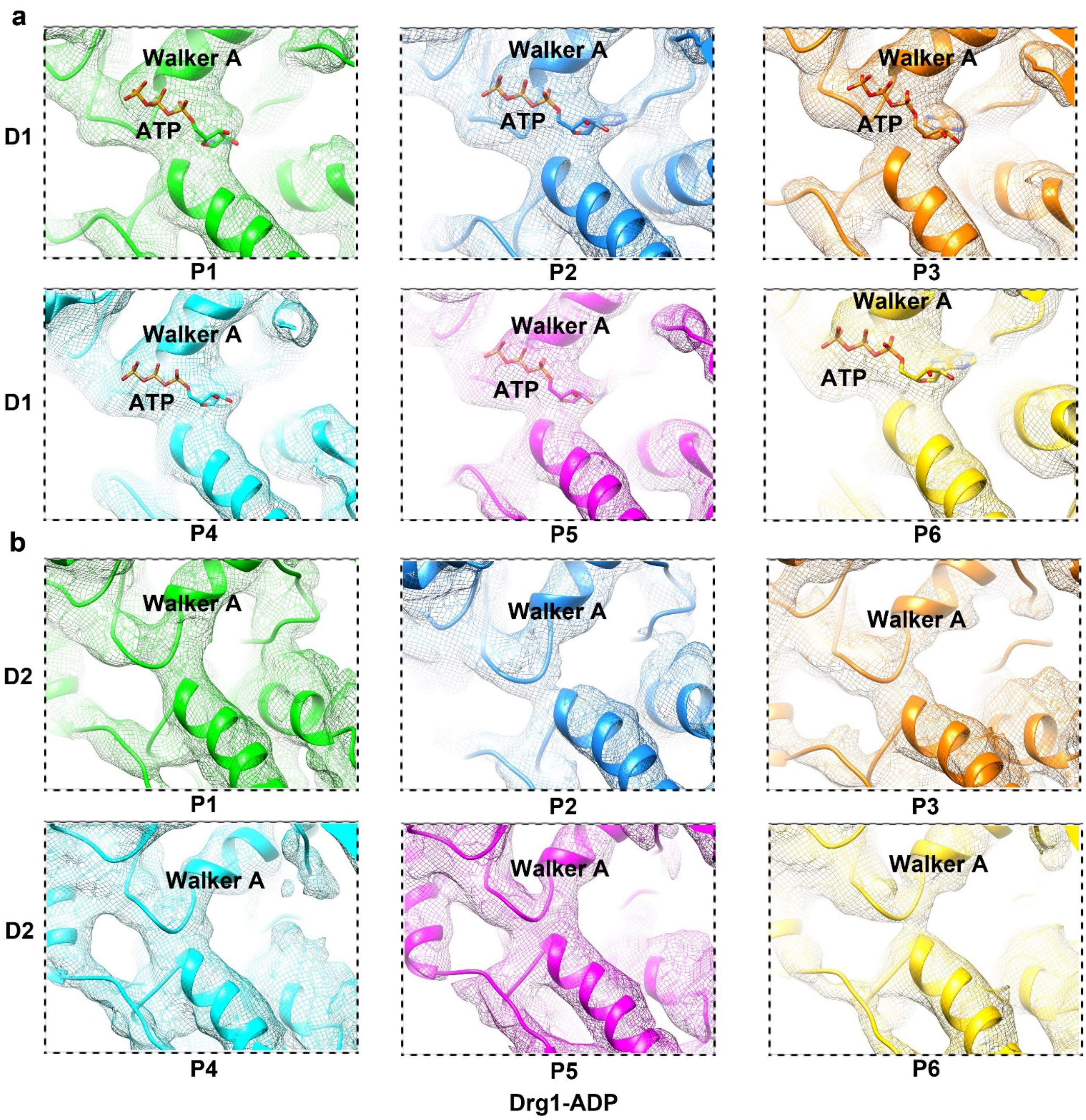

Supplementary Fig. 7 Nucleotide binding states of the 12 ATPase sites in a representative structure of the Drg1-ADP dataset.

a, ATP occupation of the six ATPase sites in the D1 ring. The atomic model and cryo-EM map are superimposed and color-coded for different protomers.

b, Empty pockets of the six ATPase sites in the D2 ring. The maps show that there is no obvious nucleotide binding to the pockets of the D2 domains.

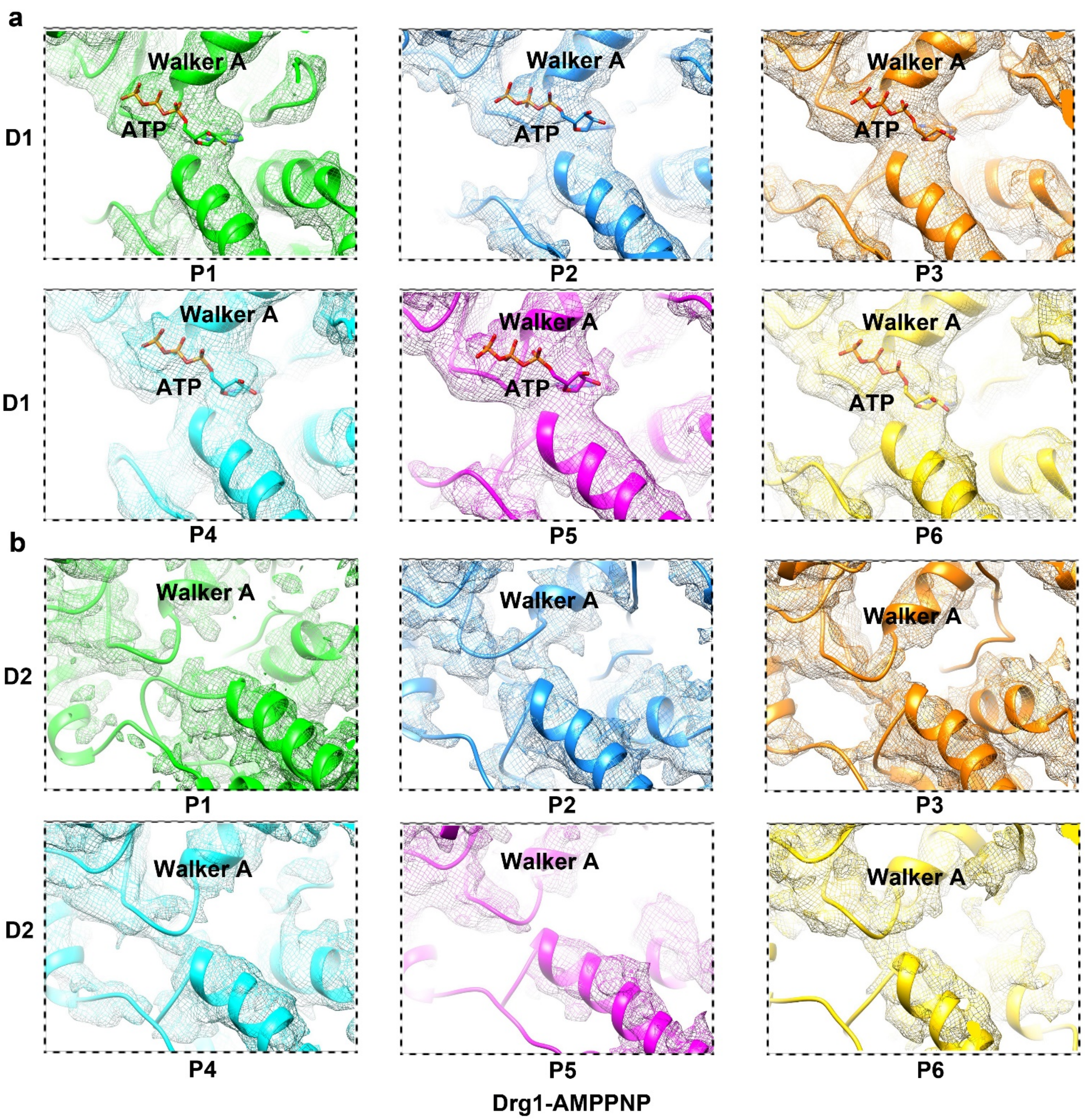

Supplementary Fig. 8 Nucleotide binding states of the 12 ATPase sites in a representative structure of the Drg1-ADP-AMPPNP dataset.

a, ATP occupation of the six ATPase sites in the D1 ring. The atomic model and cryo-EM map are superimposed and color-coded for different protomers. The map offers no distinction between ATP and AMPPNP.

b, Empty pockets of the six ATPase sites in the D2 ring. The maps show that there is no obvious nucleotide binding to the pockets of the D2 domains.

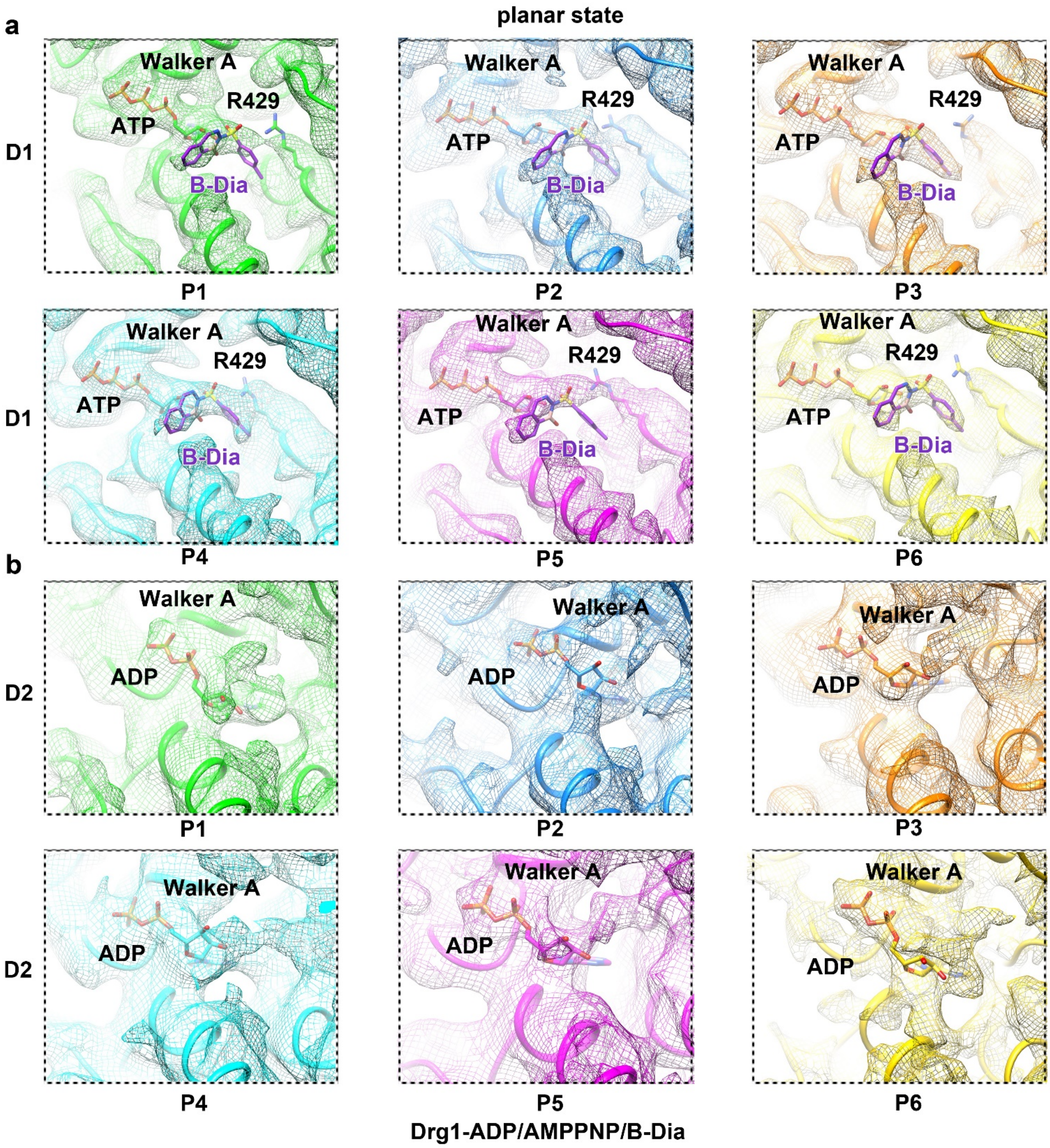

Supplementary Fig. 9 Nucleotide binding states of the 12 ATPase sites in the planar hexamer of the Drg1-ADP/AMPPNP/B-Dia dataset.

a, ATP and B-Dia occupation of the six ATPase sites in the D1 ring. The atomic model and cryo-EM map are superimposed and color-coded for different protomers.

b, Nucleotide occupation of the six ATPase sites in the D2 ring. The maps show that the six pockets of the D2 domains are all occupied. Fitting of ADP and ATP suggests a better fit for ADP in these sites. In addition, residual densities, very weak though, could be detected in expected B-Dia locations in the pockets of P1 and P6 (when displayed at low contour level).

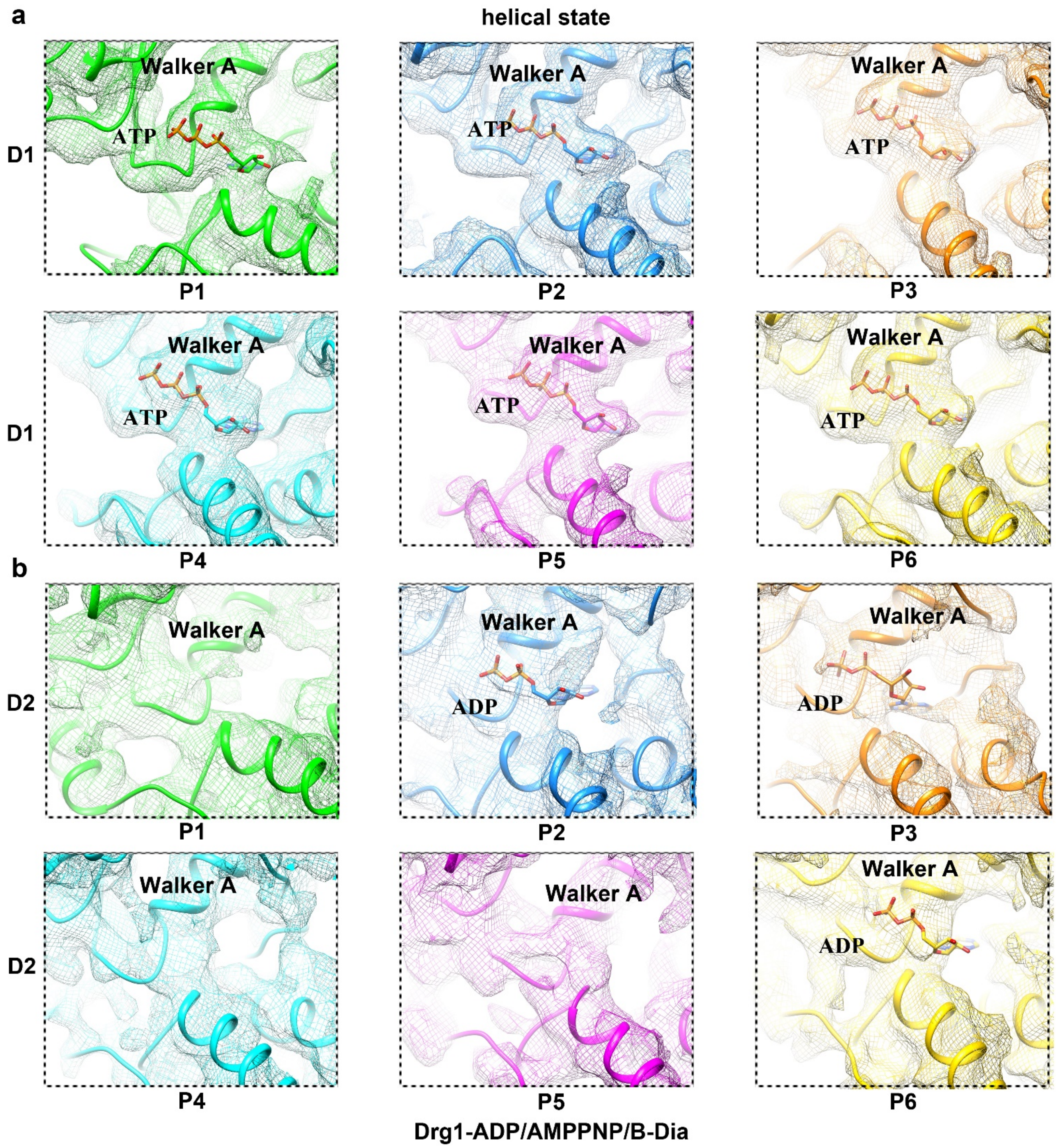

Supplementary Fig. 10 Nucleotide binding states of the 12 ATPase sites in the helical hexamer of the Drg1-ADP/AMPPNP/B-Dia dataset.

a, ATP occupation of the six ATPase sites in the D1 ring. The atomic model and cryo-EM map are superimposed and color-coded for different protomers.

b, Nucleotide occupation of the ATPase sites in the D2 ring. The local densities of the D2 pockets are not well ordered. The map suggests a presence of ADP in three D2 pockets, P2, P3 and P6.

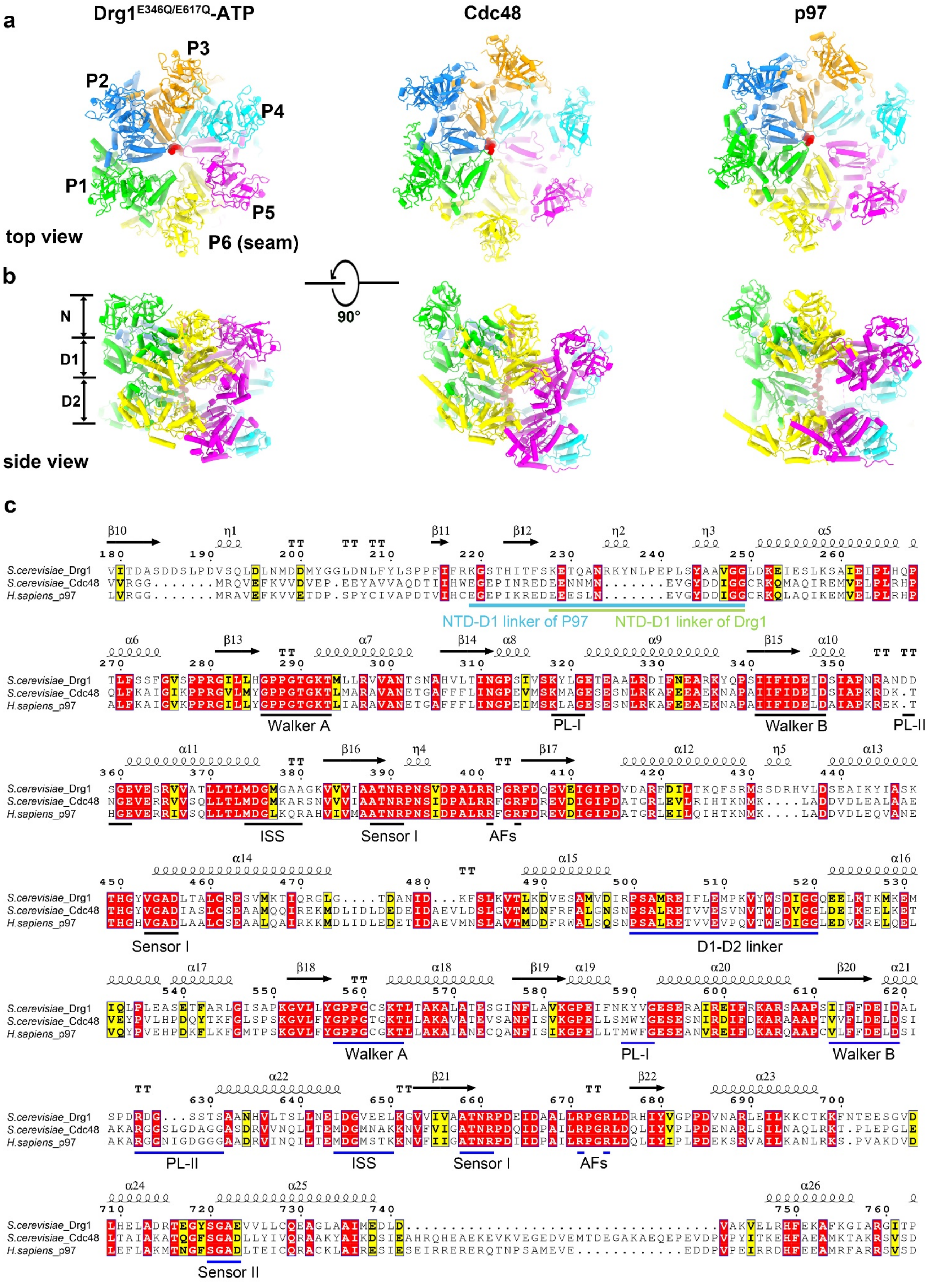

Supplementary Fig. 11 Structural comparison of *S. cerevisiae* Drg1, Cdc48 and *H. sapiens* p97 hexamers.

a, The atomic models of the Drg1, Cdc48 (PDB code: 6OPC) and p97 (PDB code: 7LN6) hexamers viewed from NTD.

b, The side view of Drg1, Cdc48 and p97 hexamers.

c, sequence alignment of Drg1, Cdc48 and p97.

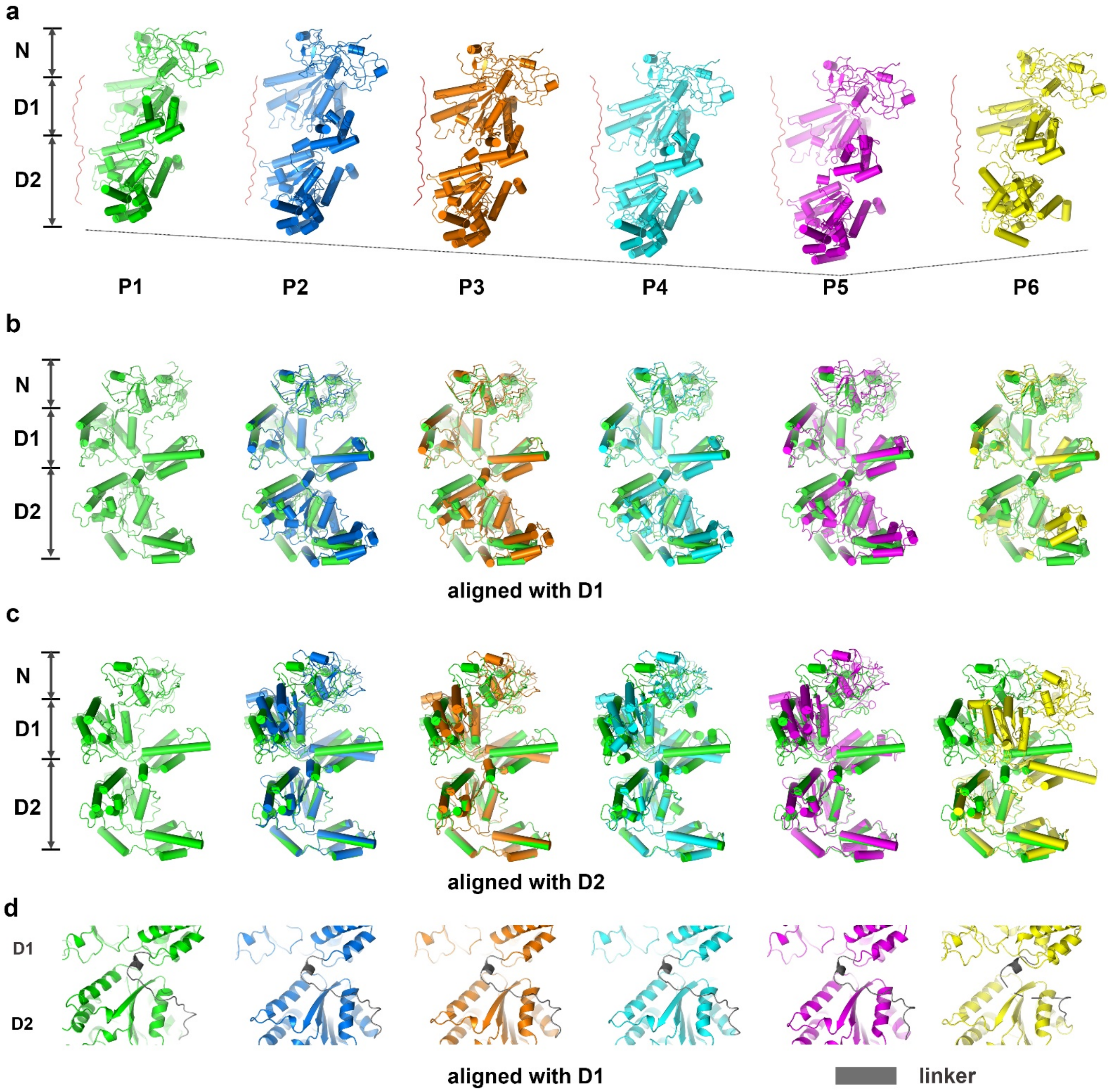

Supplementary Fig. 12 Structural comparison of the protomers from the Drg1$^{E346Q/E617Q}$-ATP complex.
a, Side-by-side comparison of the six protomers (P1 to P6). The view of each protomer was generated by consecutive 60º rotations around the substrate.
b, Pair-wise comparison of P1 with other five protomers using the D1 domain as reference. The NTD and D1 together show relative rigidity in all protomers. In contrast, the D2 domains could be grouped in three conformational groups, represented by P1, P2-P5, and P6.
c, Pair-wise comparison of P1 with other five protomers using the D2 domain as reference. Similar to b, the six protomers fall into three conformation groups according to the orientation of the D1 domain relative to the D2 domain.
d, Conformations of the D1-D2 linkers in the six protomers.

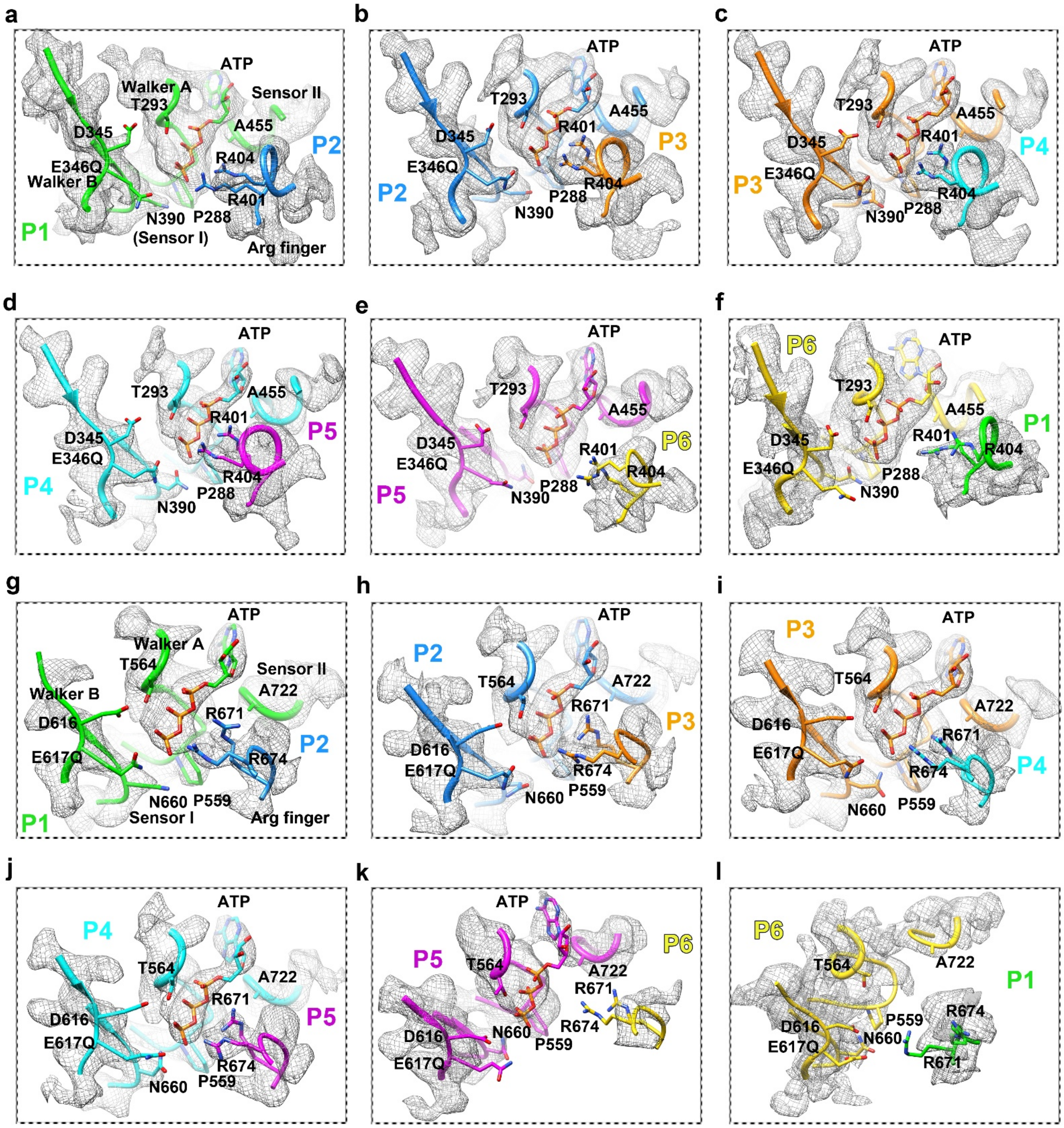

Supplementary Fig. 13 Atomic configuration of the 12 nucleotide binding sites in the Drg1$^{E346Q/E617Q}$-ATP complex.

a-f, Zoom-in views of the six nucleotide binding pockets in the D1 domains. Both the models and cryo-EM maps are shown. Selected residues of Walker A, Walker B, Sensor I, Sensor II and Arginine Finger are shown. All the six sites are occupied by ATP.

g-l, Zoom-in views of the six nucleotide binding pockets in the D2 domains. The sites of P1 to P5 are occupied by ATP. The pocket of P6 is empty.

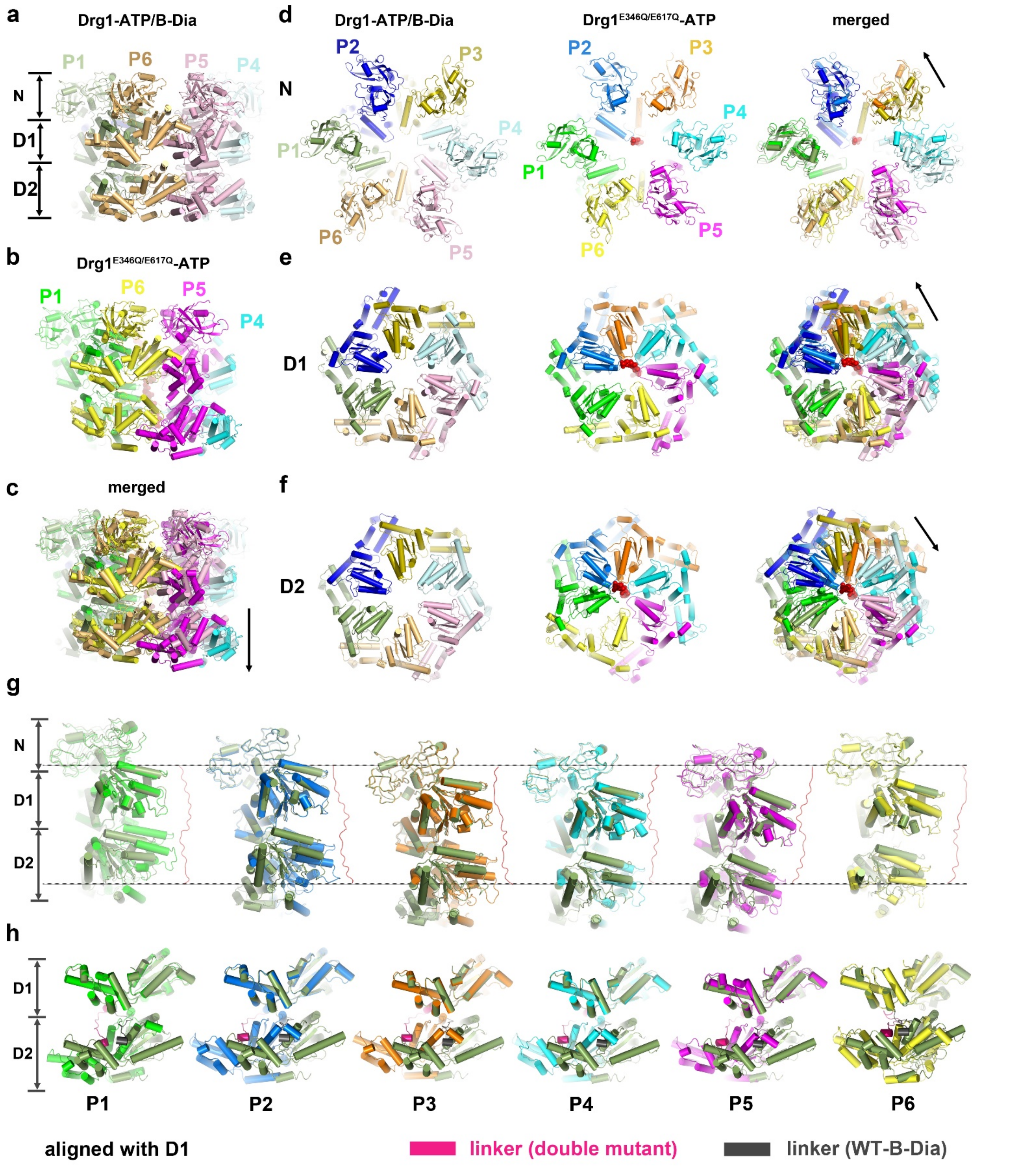

Supplementary Fig. 14 Structural comparison of the Drg1-ATP/B-Dia and Drg$^{E346Q/E617Q}$-ATP complexes.

a-c, Structural comparison of the Drg1-ATP/B-Dia and Drg1$^{E346Q/E617Q}$-ATP hexamers. Side views of the Drg1-ATP/B-Dia hexamer **(a)**, the Drg1$^{E346Q/E617Q}$-ATP hexamer **(b)** and the superimposed models of these two structures **(c)** are shown. The two models were aligned using one of their D1 domains as reference. Specifically, since the Drg1-ATP/B-Dia hexamer display a near six-fold symmetry, the D1 domain of one of its subunits was used as reference of alignment to the D1 domain of P1 from the Drg$^{E346Q/E617Q}$-ATP complex. **d-f**, Similar as **a-c**, for the comparisons of the NTD, D1 and D2 rings. Domain movements are indicated by arrows.

g, Protomer comparison between the Drg1$^{E346Q/E617Q}$-ATP and Drg1-ATP/B-Dia complexes. The six protomers of the Drg1$^{E346Q/E617Q}$-ATP hexamer (P1 to P6) are aligned with a typical subunit (forest green) of the Drg1-

ATP/B-Dia hexamer using the D1 domain as reference. According to the orientation of the D2 domain relative to the D1 domain, the conformation of the protomer of the Drg1-ATP/B-Dia hexamer is different from those of the Drg1$^{E346Q/E617Q}$-ATP hexamer.
h, Similar to g, but displayed with a rotation to highlight that the protomer of the Drg1-ATP/B-Dia hexamer is conformationally distinct from all the six protomers of the Drg1$^{E346Q/E617Q}$-ATP hexamer. A landmark used for comparison is the linker between the D1 and D2 domain. As shown, this linker (containing a short helix) is in sharply different positions between the protomer of the Drg1-ATP/B-Dia hexamer and the six protomers of the Drg1$^{E346Q/E617Q}$-ATP hexamer.

Supplementary Table 1. Statistics of data collection, processing and model validation of Drg1$^{E346Q/E617Q}$-ATP and Drg1-ATP/B-Dia dataset.

**Data collection and processing**		
Dataset	Drg1$^{E346Q/E617Q}$ -ATP	Drg1-ATP/Dia
EMDB/PDB code	32396/7WBB	32403/7WD3
Electron microscope	Titan Krios	Titan Krios
Voltage (KV)	300	300
Electron detector	K2	K2
Magnification	130,000X	130,000X
Electron dose (e-/Å^2)	64	64
Pixel size (Å)	1.057	1.052
Defocus range (μm)	-1.5 to -2.5	-1.0 to -2.5
Total micrographs	4,460	4,773
Initial particle (no.)	1,064,262	1,526,542
Final particle (no.)	152,973	348,781
Symmetry imposed	C1	C1 and C6
Map resolution (Å)	3.6 Å	3.8 Å (C1)<br>3.5 Å (C6)
**Refinement**		
Map sharping B-factor(Å^2)	-30	-100 (C6)
FSC threshold	0.143	0.143
Map resolution range (Å)	~3.2 - 6.0 Å	~3.0 - 5.0 Å (C6)
Peptide chains	7	6
Residues	4,357	4,338
Number of atoms	33,682	33,906
Ligands	ATP (11)	ATP-Dia (12)
**R.m.s. deviations**		
Bonds length (Å)	0.002	0.002
Bonds angles (^{0})	0.500	0.548
**Ramachandran plot**		
Favored (%)	94.68	92.86
Outliers (%)	0.35	0.25
Allowed (%)	4.97	6.88
**Validation**		
Molprobity score	1.84	1.93
Clashscore	8.68	8.80
Rotamer outliers (%)	0	0

Supplementary Table 2. Statistics of data collection and image processing of the Drg1-ADP, Drg1-AMPPNP and Drg1-ADP/AMPPNP/B-Dia datasets.

**Data collection and processing**				
Datasets	Drg1-ADP	Drg1-AMPPNP	Drg1-ADP/AMPPNP/B-Dia	
Electron microscope	Titan Krios	Titan Krios	Titan Krios	
Voltage (KV)	300	300	300	
Electron detector	K2	K2	K2	
Magnification	130,000X	130,000X	130,000X	
Electron dose (e-/Å^2)	64	80	64	
Pixel size (Å)	1.052	1.057	1.052	
Defocus range (μm)	-1.0 to -2.5	-1.0 to -2.5	-1.0 to -2.5	
Total micrographs	3,681	5,587	5,020	
Initial particle (no.)	1,593,028	1,823,806	2,205,661	
Final particle (no.)	51,928	285,167	148,413	88,912
Symmetry imposed	C1 and C6	C1	C1	C1
Map resolution (Å)	5.9 Å (C1)<br>4.4 Å (C6)	5.6 Å	4.3 Å<br>(planar)	5.9 Å<br>(helical)
EMDB/PDB code	32397/7YKK	32399/7YKL	32402/7YKZ	32400/7YKT
**Refinement**				
Map sharping B-factor (Å^2)	-100 (C1)	-334	-100	-50
Fourier shell correlation	0.143	0.143	0.143	0.143
Map resolution range (Å)	~4.0 - 7.0 Å	~4.0 - 7.5 Å	~4.0 - 6.5 Å	~4.5 - 7.5 Å
Peptide chains	6	6	6	6
Residues	4,410	4,410	4,404	4,410
Number of atoms	33,836	33,880	34,146	34,044
Ligands	ATP (6)	ATP (6)	ATP-Dia (6)<br>ADP (6)	ATP (6)<br>ADP (3)
**R.m.s. deviations**				
Bonds length (Å)	0.003	0.003	0.003	0.002
Bonds angles (^{0})	0.746	0.873	0.72	0.678
**Ramachandran plot**				
Favored (%)	89.81	89.23	89.60	89.17
Outliers (%)	1.3	1.21	0.98	1.14
Allowed (%)	8.89	9.56	9.41	9.69
**Validation**				
Molprobity score	2.24	2.24	2.20	2.21
Clashscore	15.13	14.49	13.55	13.29
Rotamer outliers (%)	0.03	0	0	0
