# Supplementary material for: Structural dynamics of AAA + ATPase Drg1 and mechanism of benzo-diazaborine inhibition
Source: Nat Commun. 2022 Nov 9;13:6765. doi: 10.1038/s41467-022-34511-2 (PMC9646744; doi:10.1038/s41467-022-34511-2)
Supplement: Supplementary file 3 — Reporting Summary [file 41467_2022_34511_MOESM3_ESM.pdf]

Corresponding author(s): Ning Gao

Last updated by author(s): Oct 18, 2022

# Reporting Summary

Nature Portfolio wishes to improve the reproducibility of the work that we publish. This form provides structure for consistency and transparency in reporting. For further information on Nature Portfolio policies, see our Editorial Policies and the Editorial Policy Checklist.

## Statistics

For all statistical analyses, confirm that the following items are present in the figure legend, table legend, main text, or Methods section.

| n/a | Confirmed | |
|---|---|---|
| ☐ | ☒ | The exact sample size (*n*) for each experimental group/condition, given as a discrete number and unit of measurement |
| ☐ | ☒ | A statement on whether measurements were taken from distinct samples or whether the same sample was measured repeatedly |
| ☒ | ☐ | The statistical test(s) used AND whether they are one- or two-sided<br>*Only common tests should be described solely by name; describe more complex techniques in the Methods section.* |
| ☒ | ☐ | A description of all covariates tested |
| ☒ | ☐ | A description of any assumptions or corrections, such as tests of normality and adjustment for multiple comparisons |
| ☒ | ☐ | A full description of the statistical parameters including central tendency (e.g. means) or other basic estimates (e.g. regression coefficient) AND variation (e.g. standard deviation) or associated estimates of uncertainty (e.g. confidence intervals) |
| ☒ | ☐ | For null hypothesis testing, the test statistic (e.g. *F*, *t*, *r*) with confidence intervals, effect sizes, degrees of freedom and *P* value noted<br>*Give P values as exact values whenever suitable.* |
| ☒ | ☐ | For Bayesian analysis, information on the choice of priors and Markov chain Monte Carlo settings |
| ☒ | ☐ | For hierarchical and complex designs, identification of the appropriate level for tests and full reporting of outcomes |
| ☒ | ☐ | Estimates of effect sizes (e.g. Cohen's *d*, Pearson's *r*), indicating how they were calculated |

*Our web collection on statistics for biologists contains articles on many of the points above.*

## Software and code

Policy information about availability of computer code

| | |
|---|---|
| Data collection | Cryo-EM images were collected with FEI Titan Krios TEM (Gatan K2 summit camera) by using Serial EM 3.8.7. |
| Data analysis | Motion correction was done using MotionCor2. CTF parameters were estimated by Gctf (Zhang, 2016). 2D, 3D classification and 3D refinement were done using Relion 3.0/3.1. Local resolution map was computed by Relion. The predicted 3D model from I-TASSER was as the initial template. The modelling was facilitated by the secondary structure prediction using PSIPRED. Model adjusted by using UCSF ChimeraX-1.2.5, Chimera-1.14, model building was done by Coot-0.8.7. Model refinement was done using Phenix-1.14 (https://www.phenix-online.org/). Model validation was calculated by MolProbity-4.2 (http://molprobity.manchester.ac.uk/). |

For manuscripts utilizing custom algorithms or software that are central to the research but not yet described in published literature, software must be made available to editors and reviewers. We strongly encourage code deposition in a community repository (e.g. GitHub). See the Nature Portfolio guidelines for submitting code & software for further information.

## Data

Policy information about availability of data

All manuscripts must include a data availability statement. This statement should provide the following information, where applicable:

- Accession codes, unique identifiers, or web links for publicly available datasets
- A description of any restrictions on data availability
- For clinical datasets or third party data, please ensure that the statement adheres to our policy

The cryo-EM maps (C1 symmetry) of the Drg1 hexamers from the datasets of Drg-ADP, Drg1-AMPPNP, Drg1-ADP/AMPPNP/benzo-diazaborine (helical conformation), Drg1-ADP/AMPPNP/benzo-diazaborine (planar conformation) and Drg1-ATP/benzo-diazaborine have been deposited in the EMDB (https://www.ebi.ac.uk/pdbe/emdb), with accession codes EMD-32397, EMD-32399, EMD-32400, EMD-32402, and EMD-32403, respectively. The corresponding atomic

models have been deposited in the PDB (https://www.ebi.ac.uk/pdbe/emdb) with the accession codes PDB-7YKK, PDB-7YKL, PDB-7YKT, PDB-7YKZ, and PDB-7WD3. The cryo-EM map and atomic coordinates of the Drg1 hexamer from the Drg1 mutant sample (E346Q/E617Q) have been deposited in the EMDB and PDB with accession codes EMDB-32396 and PDB-7WBB, respectively.

# Field-specific reporting

Please select the one below that is the best fit for your research. If you are not sure, read the appropriate sections before making your selection.

☒ Life sciences ☐ Behavioural & social sciences ☐ Ecological, evolutionary & environmental sciences

For a reference copy of the document with all sections, see nature.com/documents/nr-reporting-summary-flat.pdf

# Life sciences study design

All studies must disclose on these points even when the disclosure is negative.

| | |
|---|---|
| Sample size | Sample sizes were not predetermined for this study. Sample sizes for cryo-EM datasets were determined by the availability of microscope time. The number of particles used in structural determination have been shown to be sufficient. |
| Data exclusions | Regarding the cryo-EM raw micrograph screening, exclusion was done based on the quality of the images and the presence of ice contamination. Regarding the particle selection, 2D and 3D classification were used and criterion is based on the quality of resulting 2D class averages and 3D maps. |
| Replication | Multiple rounds of structural refinement have been performed and all resulted in same density maps. Every experiment reported was done at least three times with consistent results. |
| Randomization | Randomization is not applicable for the experiments involving cryo-EM. |
| Blinding | Blinding is not relevant to this study. |

# Reporting for specific materials, systems and methods

We require information from authors about some types of materials, experimental systems and methods used in many studies. Here, indicate whether each material, system or method listed is relevant to your study. If you are not sure if a list item applies to your research, read the appropriate section before selecting a response.

## Materials & experimental systems

| n/a | Involved in the study | |
|---|---|---|
| ☒ | ☐ | Antibodies |
| ☒ | ☐ | Eukaryotic cell lines |
| ☒ | ☐ | Palaeontology and archaeology |
| ☒ | ☐ | Animals and other organisms |
| ☒ | ☐ | Human research participants |
| ☒ | ☐ | Clinical data |
| ☒ | ☐ | Dual use research of concern |

## Methods

| n/a | Involved in the study | |
|---|---|---|
| ☒ | ☐ | ChIP-seq |
| ☒ | ☐ | Flow cytometry |
| ☒ | ☐ | MRI-based neuroimaging |
